# Outcome predictors in COVID-19: An analysis of emergent systemic inflammation indices in Mexican population

**DOI:** 10.3389/fmed.2022.1000147

**Published:** 2022-10-21

**Authors:** Ilse Adriana Gutiérrez-Pérez, Ivette Buendía-Roldán, Gloria Pérez-Rubio, Leslie Chávez-Galán, Rafael de Jesus Hernández-Zenteno, Hiram Aguilar-Duran, Ingrid Fricke-Galindo, Oscar Zaragoza-García, Ramcés Falfán-Valencia, Iris Paola Guzmán-Guzmán

**Affiliations:** ^1^HLA Laboratory, Instituto Nacional de Enfermedades Respiratorias Ismael Cosío Villegas, Mexico City, Mexico; ^2^Faculty of Chemical-Biological Sciences, Universidad Autónoma de Guerrero, Chilpancingo, Mexico; ^3^Translational Research Laboratory on Aging and Pulmonary Fibrosis, Instituto Nacional de Enfermedades Respiratorias Ismael Cosio Villegas, Mexico City, Mexico; ^4^Laboratory of Integrative Immunology, Instituto Nacional de Enfermedades Respiratorias Ismael Cosio Villegas, Mexico City, Mexico; ^5^COPD Clinic, Instituto Nacional de Enfermedades Respiratorias Ismael Cosío Villegas, Mexico City, Mexico

**Keywords:** systemic inflammation, biomarkers, invasive mechanic ventilation, outcome, severity, non-survival, COVID-19

## Abstract

**Introduction:**

The systemic viral disease caused by the SARS-CoV-2 called coronavirus disease 2019 (COVID-19) continues to be a public health problem worldwide.

**Objective:**

This study is aimed to evaluate the association and predictive value of indices of systemic inflammation with severity and non-survival of COVID-19 in Mexican patients.

**Materials and Methods:**

A retrospective study was carried out on 807 subjects with a confirmed diagnosis of COVID-19. Clinical characteristics, acute respiratory distress syndrome (ARDS), severity according to PaO_2_/FiO_2_ ratio, invasive mechanical ventilation (IMV), and non-survival outcome were considered to assess the predictive value and the association of 11 systemic inflammatory indices derived from hematological parameters analyzed at the hospital admission of patients. The receiver operating characteristics curve was applied to determine the thresholds for 11 biomarkers, and their prognostic values were assessed *via* the Kaplan-Meier method.

**Results:**

26% of the studied subjects showed COVID-19 severe (PaO_2_/FiO_2_ ratio ≤ 100), 82.4% required IMV, and 39.2% were non-survival. The indices NHL, NLR, RDW, dNLR, and SIRI displayed predictive values for severe COVID-19 and non-survival. NHL, SIRI, and NLR showed predictive value for IMV. The cut-off values for RDW (OR = 1.85, *p* < 0.001), NHL (OR = 1.67, *p* = 0.004) and NLR (OR = 1.56, *p* = 0.012) were mainly associated with severe COVID-19. NHL (OR = 3.07, *p* < 0.001), AISI (OR = 2.64, *p* < 0.001) and SIRI (OR = 2.51, *p* < 0.001) were associated with IMV support, while for non-survival the main indices associated were NHL (OR = 2.65, *p* < 0.001), NLR (OR = 2.26, *p* < 0.001), dNLR (OR = 1.92, *p* < 0.001), SIRI (OR = 1.67, *p* = 0.002) and SII (OR = 1.50, *p* = 0.010). The patients with an RDW, PLR, NLR, dNLR, MLR, SII, and NHL above the cut-off had a survival probability of COVID-19 50% lower, with an estimated mean survival time of 40 days.

**Conclusion:**

The emergent systemic inflammation indices NHL, NLR, RDW, SII, and SIRI have a predictive power of severe COVID-19, IMV support, and low survival probability during hospitalization by COVID-19 in Mexican patients.

## Introduction

The disease caused by the SARS-CoV-2, which was named coronavirus disease in 2019 (COVID-19), continues to be a public health problem worldwide. In Mexico, from the first identified case on February 27, 2020, until July 11, 2022, there have been 6,152,924 confirmed cases and 325,928 deaths related to COVID-19 (1, 2). Organ dysfunction, pneumonia, respiratory failure, and acute respiratory distress syndrome (ARDS) (3, 4) are some of the complications of COVID-19 that impact intensive care unit (ICU) need and invasive mechanical ventilation (IMV) support and are, in turn, associated with high mortality (5, 6). It has been described that 90% of patients with COVID-19 require IMV and are intubated within 24 h of ICU admission, and about 50% of them die (7).

The hyperinflammatory process caused by COVID-19 has been associated with severity and death due to dysregulation of the immune response to SARS-CoV-2 infection (8). Among the inflammatory markers, elevated serum levels of C-reactive protein (CRP), ferritin, D-dimer, procalcitonin, as well as cytokines (IL-10, IL-6, IL-8), and changes in CD4+ T-cell, CD8+ T-cell, as well as plasmablast counts, have been found to correlate with increased severity in patients hospitalized with COVID-19 (9). Furthermore, it has been observed that there are significant changes in the proportions of white, red, and platelet cells in patients with severe and non-severe COVID-19 (10). In this context, the presence of pronounced lymphopenia and elevated neutrophil/lymphocyte ratio (NLR), platelet/lymphocyte ratio (PLR), and monocyte/lymphocyte ratio (MLR) have been reported to be associated with severity and death from COVID-19 (11, 12). In one study, NLR and derived NLR (dNLR) indices were identified as markers of systemic inflammation predictive of poor survival and severe COVID-19 (13).

On the other hand, new markers of systemic inflammation derived from the analysis of the hematological series have been described. In a population from Italy, the systemic immune-inflammation index (SII) was the most important prognostic biomarker for survival in patients infected with SARS-CoV-2 (14). In contrast, the aggregate index of systemic inflammation (AISI) was an important predictor of severity and ICU admission in patients with COVID-19 (15).

Hematological indices are currently considered inflammation and clinical prognosis biomarkers of viral pneumonia. However, not all parameters have been significantly associated between populations, in addition to the variability reported when defining the cut-off values associated with severity or death by COVID-19, so it is essential to evaluate and define the indices of clinical prognostic and diagnostic utility as well as to determine the cut-off values that define risk among the Mexican population. This study aims to evaluate the predictive value and association of systemic inflammation indices with severity and mortality in Mexican patients with COVID-19.

## Materials and methods

### Study design and samples collection

A retrospective study was carried out in a cohort of 807 hospitalized patients from the Instituto Nacional de Enfermedades Respiratorias Ismael Cosio Villegas (INER; National Institute for Respiratory Diseases), Mexico City, between October 1, 2020, and December 31, 2021. The patients were diagnosed with COVID-19 according to international guidelines. All subjects tested positive on the nasopharyngeal swab for detecting SARS-CoV-2 by reverse transcriptase-polymerase chain reaction (RT-PCR). The study protocol was approved by the Institutional Ethical Research and Investigation Committees (approval number C53-20), and all procedures were performed following the Helsinki Declaration.

Sociodemographic data, symptoms, clinical data, and the presence of comorbidities were documented in electronic medical records using a standardized data collection form. The assessment results of blood samples obtained from the patients during hospital admission were considered to evaluate and define the systemic inflammation indices. The hematological series were evaluated in a hemocytometer [UniCel DxH 800 Coulter Cellular Analysis System] using a peripheral blood sample obtained into a tube with EDTA [BD Vacutainer 368159, Franklin Lakes, NJ, USA] as an anticoagulant.

Clinical outcomes related to clinical symptoms such as disease severity, IMV, and survival or death were registered during the hospitalization stay. The exclusion criteria were patients under 18 years old or having missing laboratory data, as well as patients not having all the information of interest in the electronic files of medical records. Those who did not provide a blood sample were also excluded.

### Definitions

In the present study, the patients were divided based on the clinical severity of the COVID-19, IMV, and clinical outcome. The severity of COVID-19 was defined according to the PaO_2_/FiO_2_ ratio at hospital admission as follows: mild (>200), moderate (100–200), and severe (≤100) (16). The support of IMV was considered from admission to the hospital or during the following hospitalization time. Death’s outcome was considered non-survival and discharged by improvement as survival.

Eleven systemic inflammation indices were defined from blood samples collected at hospital admission in this study as follows:

• Red Blood Cell Distribution Width (RDW) = ratio of the standard deviation of red blood cell volume and mean corpuscular volume × 100.
• Neutrophil to lymphocyte ratio (NLR) = absolute neutrophil count (ANC)/absolute lymphocyte count (ALC).
• Monocyte to lymphocyte ratio (MLR) = absolute monocyte count (AMC)/ALC.
• Basophil-to-lymphocyte ratio (BLR) = absolute basophil count (ABC)/ALC.
• Eosinophil-lymphocyte ratio (ELR) = absolute eosinophil count (AEC)/ALC.
• Platelet to lymphocyte ratio (PLR) = absolute platelet count (APC)/ALC.
• Derived NLR (dNLR) = ANC/(WBC - ANC).
• Systemic immune-inflammation index (SII) = absolute platelets count (APC) × NLR.
• Systemic inflammation response index (SIRI) = (ANC × AMC)/ALC.
• Aggregate index of systemic inflammation (AISI) = (ANC × AMC × APC)/ALC.
• Neutrophil-to-hemoglobin and lymphocyte (NHL) = ANC (Hb × ALC).

### Statistical analysis

Categorical variables were presented as frequencies and percentages, and the differences between groups were assessed using a chi-square test. Quantitative data were presented as median with 5th and 95th percentile ranges, and a Mann-Whitney *U* test or Kruskal Wallis was made to assess differences between groups. The predictive values of eleven hematological indices for severity, IMV, and non-survival were determined by analyzing a receiver operating characteristic (ROC) curve and the area under the ROC curve (AUC). In addition, the cut-off values were defined using sensitivity and specificity. A regression logistic multivariate analysis was performed to evaluate the association between the cut-off values for systemic inflammation indices and the presence of severity, IMV, and non-survival. The survival curves were analyzed using the Kaplan-Meier method and compared using the Log-rank test. Data were processed using STATA v15.0 and GraphPad Prism v.8.4 software for windows. *p*-values < 0.05 were considered statistically significant.

## Results

### Demographic and clinical characteristics

This study included data from 807 patients, 279 women, and 528 men. The median age of hospitalized patients was 59 years old. However, the prevalent group was significantly older and men. The presence of overweight (*p* = 0.004) and tobacco smoking (*p* < 0.001) was significantly higher in men in comparison to women, while in women, the frequencies of previous chronic respiratory disease (PCRD) (*p* = 0.009), type 2 diabetes (T2D), and hypertension were higher (Table 1).

**TABLE 1:** Demographic and clinical characteristics in patients with COVID-19.

Characteristics	Total (*n* = 807)	Women (*n* = 279)	Men (*n* = 528)	*p-*value
**Demographics**				
Age, years, median (P_5_–P_95_)^a^	59 (35–81)	62 (35–83)	59 (35–80)	0.002
Age category, *n* (%)^b^				0.003
≤50 years	196 (24.3)	58 (20.8)	138 (26.2)	
51–65 years	352 (43.6)	110 (39.4)	242 (45.8)	
≥65 years	259 (39.1)	111 (39.8)	148 (28.0)	
BMI, kg/m^2^, median (P_5_-P_95_)^a^	28.9 (22.9–40.9)	30.5 (23.0–42.2)	28.4 (22.8–39.2)	<0.001
BMI category, *n* (%)^b^				0.004
Normal weight	126 (15.6)	37 (13.3)	89 (16.9)	
Overweight	329 (40.8)	98 (35.1)	231 (43.7)	
Obesity	352 (43.6)	144 (51.6)	208 (39.4)	
**Comorbidities presence**				
Tobacco smoking, yes, *n* (%)^b^	246 (30.5)	50 (17.9)	196 (37.1)	<0.001
T2D, yes, *n* (%)^b^	232 (28.8)	91 (32.6)	141 (26.7)	0.080
Hypertension, yes, *n* (%)^b^	304 (37.7)	117 (41.9)	187 (35.5)	0.072
PCRD, yes, *n* (%)^b^	60 (7.5)	30 (10.7)	30 (5.7)	0.009
**Clinical characteristics**				
Symptoms onset days, median (P_5_-P_95_)^a^	10 (4–21)	9 (3–20)	10 (4–21)	0.002
Hospitalization days, median (P_5_-P_95_)^a^	22 (8–68)	21 (8–64)	22 (9–68)	0.436
IMV, yes, *n* (%)^b^	665 (82.4)	217 (77.8)	448 (84.9)	0.012
PaO_2_/FiO_2_ ratio, *n* (%)^b^				0.541
>200	138 (17.1)	45 (16.1)	93 (17.6)	
101–200	459 (56.9)	155 (55.6)	304 (57.6)	
≤100	210 (26.0)	79 (28.3)	131 (24.8)	
Outcome, *n* (%)^b^				0.120
Survival	491 (60.8)	180 (64.5)	311 (58.9)	
Non-survival	316 (39.2)	99 (35.5)	217 (41.1)	

BMI, body mass index; IMV, invasive mechanic ventilation; PCRD, previous chronic respiratory disease; T2D, type 2 diabetes.

^a^Data are expressed as the median and percentiles 5th–95th, compared using Mann Whitney *U*-test.

^b^Data are expressed as the n (%), compared using the Chi-square test.

*p*-value < 0.05 was considered statistically significant.

Table 1 shows that the hospital admission time was around 10 days after the onset of the symptoms. However, the admission of men was later than that of women (*p* = 0.002). 82.4% of patients required support from IMV, mainly men (*p* = 0.012). According to the PaO_2_/FiO_2_ ratio category, 26% were categorized as severe COVID-19, and 39.2% were non-survival, without differences between sexes.

**FIGURE 1:**
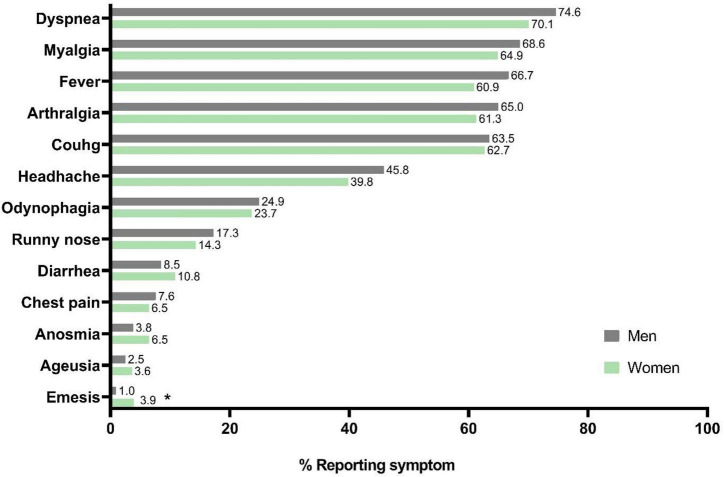
Distribution of frequencies by gender to the symptomatology of COVID-19 in Mexican patients. Statistical analyses were performed by Chi-square test. Significance was set at *p*-value < 0.05. *p = 0.004.

Figure 1 shows the clinical symptoms of COVID-19. These were similar among the sexes, and in general, the most common symptoms were dyspnea (73.1%), myalgia (67.3%), fever (64.7%), arthralgia (63.7%), and cough (63.2%). Only emesis was significantly more frequent in women than men (*p* = 0.004, 3.94 vs. 0.95%, respectively).

The comorbidities and sociodemographic data significantly associated to the PaO_2_/FiO_2_ ratio ≤ 100 were PCRD (OR = 2.01, 95% CI, 1.17–3.47, *p* = 0.011), age > 65 years old (OR = 1.63, 95% CI, 1.91–4.01, *p* < 0.001), and obesity (OR = 1.38, 95% CI, 1.00–1.89, *p* = 0.045). Only the male gender (OR = 1.60, 95% CI, 1.10–2.31, *p* = 0.013) was associated with IMV support. Finally, the principal factor associated with non-survival by COVID-19 was age > 65 years old (OR = 2.77, 95% CI, 1.91–4.01, *p* < 0.001) (Supplementary Table 1).

Leukocytosis and higher counts for neutrophils were shown according to the severity of COVID-19, IMV, and non-survival, while the parameters related to the red blood cells, as well as the platelet count, were displayed lower according to severity (Supplementary Tables 2, 3).

### Use of the optimum cut-off values of systemic inflammation indices in severity, invasive mechanical ventilation, and non-survival of COVID-19 patients

In Table 2, the hematologic indices such as SIRI, RDW, NLR, and NHL displayed a significantly higher predictive value (AUC ≥ 0.60) for severe COVID-19 (PaO_2_/FiO_2_ ≤ 100), while for IMV, eight indices, including RDW, NLR, MLR, dNLR, AISI, SII, SIRI, and NHL show predictive value (AUC ≥ 0.60). However, SIRI and NLR showed higher values (AUC ≥ 0.65). For non-survival, the leading indices were NLR, dNLR and NHL (AUC ≥ 0.60). The optimal cut-off value of each hematological index was determined, as well as the sensitivity and specificity. The NLR and NHL displayed a higher sensibility and specificity.

**TABLE 2:** Predictor values of systemic inflammation indices on severity and non-survival in COVID-19 patients.

Hematologic Indices	PaO_2_/FiO_2_ ≤ 100	IMV	Non-survival	Cut-off value	Sensibility%	Specificity%
		
	AUC (95% CI), *p-*value	AUC (95% CI), *p-*value	AUC (95% CI), *p-*value			
RDW	0.616 (0.555–0.678), <0.001	0.625 (0.571–0.608), <0.001	0.568 (0.527–0.610), 0.001	≥13.95	64.3	46.4
PLR	0.542 (0.480–0.604), 0.189	0.546 (0.493–0.599), 0.094	0.533 (0.491–0.576), 0.111	≥303	65.6	40.6
NLR	0.623 (0.562–0.683), < 0.001	0.664 (0.613–0.714), < 0.001	0.647 (0.608–0.686), < 0.001	≥11.0	73.3	50.2
MLR	0.584 (0.523–0.646), 0.008	0.621 (0.572–0.670), < 0.001	0.568 (0.526–0.610), 0.001	≥0.58	71	40
ELR	0.527 (0.464–0.589), 0.400	0.558 (0.507–0.609), 0.033	0.511 (0.469–0.553), 0.598	≥0.016	23.7	77.2
BLR	0.501 (0.438–0.565), 0.952	0.512 (0.460–0.565), 0.637	0.510 (0.468–0.552), 0.638	≥0.02	14	87.3
dNLR	0.551 (0.504–0.598), 0.028	0.639 (0.585–0.687), <0.001	0.643 (0.603–0.682), < 0.001	≥6.5	65	54.8
SII	0.590 (0.529–0.651), 0.004	0.636 (0.585–0.687), <0.001	0.588 (0.547–0.629), < 0.001	≥2,892	65	46.8
SIRI	0.606 (0.545–0.667), 0.001	0.671 (0.622–0.720), < 0.001	0.597 (0.556–0.638), < 0.001	≥4.29	72	40
AISI	0.581 (0.520–0.642), 0.011	0.643 (0.595–0.682), < 0.001	0.542 (0.500–0.584), 0.048	≥1,189	64.7	40
NHL	0.648 (0.588–0.707), <0.001	0.704 (0.656–0.752), < 0.001	0.665 (0.626–0.703), < 0.001	≥0.83	75.3	50.1

AISI, aggregate index of systemic inflammation; AUC, area under curves; BLR, basophil-to-lymphocyte ratio; dNLR, derived NLR; ELR, eosinophil-lymphocyte ratio; MLR, lymphocyte to monocyte ratio; NHL, neutrophil-to-hemoglobin and lymphocyte; NLR, neutrophil to lymphocyte ratio; PLR, platelet to lymphocyte ratio; RDW, red blood cell distribution width; SII, systemic immune-inflammation index; SIRI, systemic inflammation response index.

The AUC was analyzed by ROC curves. *p*-value < 0.05 was considered statistically significant.

We observed that the patients’ ages were statistically different between men and women (Table 1), and that some comorbidities, such as obesity, were greater in women, while tobacco smoking was more frequent in men. In a stratified analysis by sex, categorized age, tobacco smoking, and comorbidities, it was observed that the indices of systemic inflammation were associated with the severity of COVID-19, IMV, and mortality, mainly in women. According to age, the NLR, dNLR, PLR, MLR, SIRI, SII, and NHL indices were associated with severity and mortality in patients aged over 65 years. On the other hand, in tobacco smoking patients, the main predictors of severity and poor outcome of COVID-19 were NLR, dNLR, RDW, SII, and NHL, while in patients with comorbidities such as diabetes and hypertension they were mainly NLR, dNLR, MLR, PLR, SII, SIRI, and NHL (Supplementary Figures 1A–G).

**FIGURE 2:**
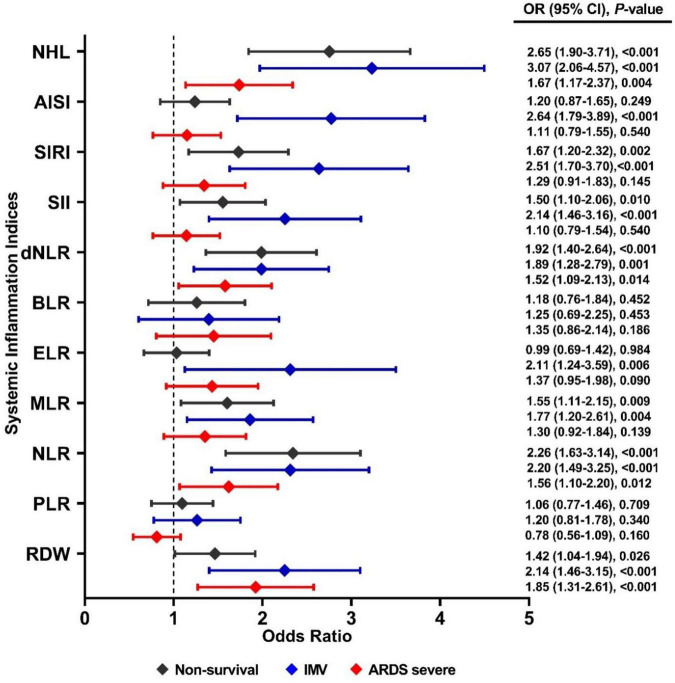
Association between systemic inflammation indices and severe COVID-19, IMV support, and non-survival by COVID-19 in Mexican patients. Statistical analyses were performed by a multivariate logistic regression model adjusted by sex and age. Significance was set at *p*-value < 0.05.

In a multivariate logistic regression model adjusted by sex and age, the RDW ≥ 13.95 (OR = 1.85, 95% CI, 1.31–2.61, *p* < 0.001), NHL ≥ 0.83 (OR = 1.67, 95% CI, 1.17–2.37, *p* = 0.004), NLR ≥ 11 (OR = 1.56, 95% CI, 1.10–2.20, *p* = 0.012) and dNLR ≥ 6.5 (OR = 1.52, 95% CI, 1.09–2.13, *p* = 0.014) were the principal indices associate to severe COVID-19. For IMV, the main indices associated were NHL ≥ 0.83 (OR = 3.07, 95% CI, 2.06–4.57, *p* < 0.001), AISI ≥ 1,189 (OR = 2.64, 95% CI, 1.79–3.89, *p* < 0.001), SIRI ≥ 4.29 (OR = 2.51, 95% CI, 1.70–3.70, *p* < 0.001), NLR ≥ 11 (OR = 2.2, 95% CI, 1.49–3.25, *p* < 0.001), and dNLR ≥ 6.5 (OR = 1.89, 95% CI, 1.28–2.79, *p* = 0.001). Meanwhile, for non-survival were NHL ≥ 0.83 (OR = 2.65, 95% CI, 1.90–3.71, *p* < 0.001), NLR ≥ 11 (OR = 2.26, 95% CI, 1.63–3.14, *p* < 0.001), dNLR ≥ 6.5 (OR = 1.92, 95% CI, 1.40–2.64, *p* < 0.001), SIRI ≥ 4.29 (OR = 1.67, 95% CI, 1.20–2.32, *p* = 0.002), and MLR (OR = 1.55, 95% CI, 1.11–2.15, *p* = 0.009) (Figure 2).

### Kaplan-Meier curves of systemic inflammation indices in the COVID-19 progression

**FIGURE 3:**
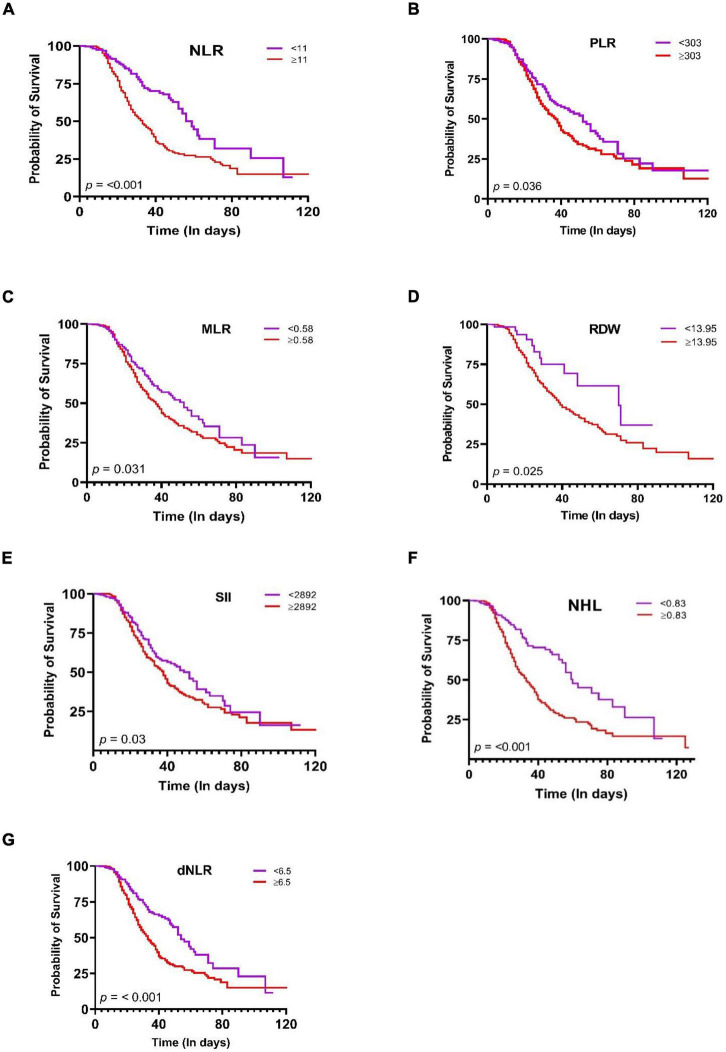
Kaplan–Meier survival curves during hospitalization of COVID-19 in Mexican patients with different cut-off values of the systemic inflammation indices. **(A)** NLR, **(B)** PLR, **(C)** MLR, **(D)** RDW, **(E)** SII, **(F)** NHL, **(G)** dNLR. Statistical analyses were performed by Kaplan-Meier method and compared using the Log-rank test. Significance was set at *p*-value < 0.05.

Finally, the survival duration was significantly different among groups (Long rank *p* < 0.05) according to cut-off points. The probability of survival of patients with a RDW ≥ 13.95 (*p* = 0.025), PLR ≥ 303 (*p* = 0.036), NLR ≥ 11, (*p* < 0.001), dNLR ≥ 6.5 (*p* < 0.001), MLR ≥ 0.58 (*p* = 0.031), SII ≥ 2,892 and NHL ≥ 0.83 (*p* < 0.001) was lower 50%, with an estimated mean survival time of 40 days (Figures 3A–G). On the other hand, the hematological indices SIRI, ELR, BLR, and AISI were not significantly related (Supplementary Figures 2A–D).

## Discussion

The main findings in this study were as follows: (i) the systemic inflammation indices NHL, NLR, SIRI, and the RDW show a high predictive value for a PaO_2_/FiO_2_ ≤ 100, IMV, and non-survival in patients with COVID-19; (ii) independently of age and sex, NHL, NLR SIRI, and MLR are associated with non-survival by COVID-19; (iii) PLR, NLR, MLR, SII, RDW, and NHL indices are associated with low survival during the hospital stay due to COVID-19.

In the early stages of SARS-CoV-2 pneumonia, a state of exudative inflammation, perivascular infiltration, presence of interalveolar multinucleated giant cells, pneumocyte hyperplasia, and intracytoplasmic viral inclusion bodies in lung tissue are established (17). Thus, cell recruitment in the lung, in addition to modifying the circulating cellular proportions, compromises the integrity of the tissue, favoring damage and the requirement for IMV. In a consecutive series of 50 cases of Italian patients with COVID-19 who presented a prolonged hospital stay and a low PaO_2_/FiO_2_ ratio, lymphopenia, and elevated C reactive protein (CRP) and lactate dehydrogenase levels were reported (18). In this study, we found that the relation between lymphocytes and other cellular phenotypes of the white and red series define indices of systemic inflammation predictive of severe COVID-19 and the support with IMV.

In cases of severe COVID-19, lymphopenia is a common feature related to immune hyperactivity. It suggests a state of systemic self-destructive inflammation, in which, although the T cell count decreases, T cells exhibit a hyperactivated phenotype (19, 20). Similarly, the effect of cytokine storm has an impact on the number, morphology, and activation of immune cells such as neutrophils, lymphocytes, monocytes, and platelets (21), and even on red blood cell parameters, as reported by Anani et al. (22) in patients hospitalized for COVID-19, who presented low hemoglobin and hematocrit levels, as well as an increase in RDW, NLR, and lymphocytes. In our study, NHL and NLR parameters were consistently the best predictors of mortality.

In hospitalized patients from Romania (23) and Italy (14), the indices NLR, MLR, SIRI, dNLR, and AISI were the main predictors of mortality. However, our study is the first to report the NHL index in COVID-19 subjects. The NHL index has recently been incorporated and used in the evaluation of inflammatory processes such as rheumatoid arthritis (24), non-muscle-invasive bladder cancer (25), and acute myocardial infarction (26), processes in which it is positively related to clinical activity and disease severity.

The presence of hemoglobin levels less than 11.6 g/dL has been evaluated as a predictor of severity of COVID-19, with a clinical prognostic specificity of 79.3%; also, low hemoglobin levels have been associated with increased mortality and the probability of ICU admission (27). In this study, we report that the NHL index represents an emerging indicator in the clinical prognosis of COVID-19. The hyperinflammatory state in COVID-19 orchestrated by cytokines such as IL-6 is one of the mechanisms explaining the presence of impaired erythropoiesis. IL-6 regulates iron homeostasis by inducing hepcidin synthesis in hepatocytes, inhibiting iron absorption in the duodenum, and sequestration of recycled iron by senescent erythrocytes (28). Zhou et al. (29) reported that elevated levels of hepcidin and serum ferritin are associated with the severity of COVID-19; thus, monitoring red formula during COVID-19 infection is critical in the prognostic evaluation of the patient.

In a model adjusted by age, comorbidities, and gender, dNLR was associated with 14.09 times higher odds of in-hospital death compared to NLR (OR = 4.14), MLR (OR = 3.29), and SIRI (OR = 3.06) (23). In this study, NHL and NLR showed a higher association and predictive power for mortality in patients with COVID-19 compared to dNLR or SIRI. Hematologic indices, including neutrophil count, show higher predictive values for non-survival. Neutrophils are the first innate immune cells recruited during antiviral immune responses and other infections, and these cells represent approximately 50–70% of all circulating leukocytes (30). This supports the relation observed in this study between severity and systemic inflammation assessed through NLR, NHL, dNLR, AISI, SIRI, and SII. Neutrophils, upon activation, trigger a destruction mechanism in which structures called neutrophil extracellular traps (NETs) are released (31). NETs are abundant at sites of acute inflammation and have been detected in tracheal aspirate, lung tissue, and plasma of patients with COVID-19 (32). Their release has been associated with tissue damage by extracellular exposure of DNA, granular proteins such as myeloperoxidase, and histones, inducing processes of apoptosis and fibrosis (33, 34). SARS-CoV-2 infection increases the levels of reactive oxygen species by activating the formation of NETs in neutrophils, conditioning an oxidizing environment (35), and causing endothelial injury and neuroinflammation through complement activation, in addition to promoting thrombus formation (36).

In a Turkish population hospitalized for COVID-19, it was observed that elevated levels of D-dimer, CRP, and NLR are predictors of severity (37); in UK patients infected with SARS-CoV-2, it has been observed that markers of inflammation such as lymphocytes < 1.5 10^9^/L, NLR, and hematocrit (<0.40 L/L men, < 0.37 L/L women) were associated with mortality within 30 days of hospitalization (38). We observed in this study an average hospitalization time of 40 days. The RDW, PLR, NLR, dNLR, MLR, SII, AISI, and NHL markers were associated with less than 50% survival during the hospital stay. Citu et al. (23) reported that the median survival days associated with altered NLR were 28.3 days, 26.5 days for dNLR, 27.7 days for MLR, and 28 days for SIRI. Our findings show an association between hematologic indices of systemic inflammation assessed during hospitalization admission and severity and outcome of COVID-19, so the values established in this study could consider patients at potential risk of poor clinical outcome and should have prompt access to the ICU and support with IMV, as suggested by Liu et al. (39) in patients ≥ 50 years and with an NLR ≥ 3.13. Therefore, the emerging hematologic indices analyzed are low-cost markers and can be used in managing SARS-CoV-2 infected patients and as biomarkers of poor clinical prognosis in COVID-19 patients.

It is important to consider the association of COVID-19 severity and non-survival with comorbidities in our population to establish comparable populations. In our study, male sex and obesity were associated with IMV support. In a population of US veterans with COVID-19, Ioannou et al. (40) reported that black race, male sex, T2D, and hypertension are associated with IMV; moreover, adulthood, male sex, coronary artery disease, T2D and obesity, nicotine use (41), and cardiovascular disease (42) are associated factors with a long time with IMV support.

As observed, male sex and older age are variables related to the use of IMV. We observed that hematologic indices of systemic inflammation such as NHL, dNLR, AISI, and SIRI were associated independently of age and sex with IMV, making them quick and easily accessible tools to screen COVID-19 patients to prevent the development of severe complications and death from COVID-19. It has been suggested that early intubation is associated with improved survival rates in patients with severe ARDS associated with COVID-19 pneumonia (43).

The possible limitations of our study are that bacterial or fungal infections are not analyzed during the hospital stay, and the use of pharmacological therapies could be related to the clinical outcome. To date, no exclusive and effective treatment for COVID-19 has been reported; therefore, it is crucial to identify and incorporate new biomarkers in clinical practice that are affordable for the different hospitals in the health sector to assist in decision making, such as early identification of possible complications during the disease and/or hospital stay.

## Conclusion

In conclusion, the emergent systemic inflammation indices NHL, NLR, dNLR, RDW, SII, and SIRI have a predictive power of severe COVID-19, IMV support, and low survival during hospitalization by COVID-19 in Mexican patients.

## Data availability statement

The original contributions presented in the study are included in the article/Supplementary material, further inquiries can be directed to the corresponding author/s.

## Ethics statement

The study protocol was approved by the Institutional Ethical Research and Investigation Committees (approval number C53-20), and all procedures were performed following the Helsinki Declaration. The patients/participants provided their written informed consent to participate in this study.

## Author contributions

IG-G and IG-P contributed to conceptualization and design of the study, performed the statistical analysis, and contributed to writing—original draft preparation. RF-V, IB-R, GP-R, HA-D, IG-P, IF-G, and OZ-G contributed to methodology. IB-R, HA-D, LC-G, and RH-Z performed the clinical evaluation of patients. IG-P contributed to data curation. RF-V and IG-G contributed to supervision. All authors contributed to manuscript revision and read and approved the submitted version.
