# Supplementary material for: Outcome predictors in COVID-19: An analysis of emergent systemic inflammation indices in Mexican population
Source: Front Med (Lausanne). 2022 Oct 21;9:1000147. doi: 10.3389/fmed.2022.1000147 (PMC9633849; doi:10.3389/fmed.2022.1000147)
Supplement: Supplementary file 1 [file Data_Sheet_1.pdf]

Supplementary Material

TABLES

Supplementary TABLE 1 | Association between comorbidities, demographic factors, and outcome in COVID-19 patients

	$PaO_2/FiO_2 \leq 100$	IMV	Non-survival
**Characteristics**	OR (95% IC), *p*-value	OR (95% IC), *p*-value	OR (95% IC), *p*-value
Sex male	0.83 (0.60-1.15), 0.281	1.6 (1.10-2.31), 0.013	1.26 (0.93-1.71), 0.120
>65 years old	1.63 (1.09-2.41), 0.016	1.22 (0.81-1.84), 0.331	2.77 (1.91-4.01), <0.001
Tobacco smoking	1.34 (0.96-1.87), 0.082	1.19 (0.79-1.78), 0.390	1.06 (0.78-1.45), 0.675
T2D	0.87 (0.61-1.24), 0.461	0.81 (0.54-1.19), 0.269	1.11 (0.81-1.52), 0.490
HAS	0.85 (0.61-1.18), 0.334	1.27 (0.87-1.87), 0.212	1.28 (0.95-1.71), 0.096
PCRD	2.01 (1.17-3.47), 0.011	0.84 (0.43-1.63), 0.618	1.50 (0.89-2.55), 0.126
Obesity	1.38 (1.0-1.89), 0.045	1.37 (0.94-1.99), 0.090	0.57 (0.42-0.76), <0.001

HAS, hypertension arterial systemic; *IMV*, invasive mechanic ventilation; *PCRD*, previous chronic respiratory disease; *T2D*, type 2 diabetes.
Statistical analyses were performed by a multivariate logistic regression model adjusted by sex and age Significance was set at a *p*-value <0.05.

Supplementary TABLE 2 | Hematological parameters and systemic inflammation indices in COVID-19 patients according to PaO_2/FiO_2 ratio category

Characteristics	PaO_2/FiO_2 ratio			
**Parameters**	**>200**	**200-101**	**≤100**	***p*-value**
WBC,$10^3/mm^3$	9.3 (5.1-17)	10 (4.8-19.3)	10.45 (5.7-19.2)	0.004
Neutrophil count, $10^3/mm^3$	8 (3.2-15.8)	8.6 (4-17.3)	9.1 (4.6-17)	0.003
Lymphocyte count, $10^3/mm^3$	0.7 (0.2-2.0)	0.6 (0.2-1.9)	0.6 (0.2-1.9)	0.010
Monocyte count, $10^3/mm^3$	0.5 (0.2-1.1)	0.5 (0.2-1.2)	0.5 (0.2-1.3)	0.873
Eosinophil, count $10^3/mm^3$	0 (0-0.3)	0 (0-0.4)	0 (0-0.4)	0.232
Basophil count, $10^3/mm^3$	0 (0-0.1)	0 (0-0.1)	0 (0-0.1)	0.103
Erythrocytes, $10^6/mm^3$	4.6 (2.94-5.45)	4.4 (3-5.5)	4.3 (2.8-5.5)	0.004
Hemoglobin, gr/dL	13.8 (9.2-16.8)	13.4 (9.1-16.8)	13 (8.6-17)	0.010
Hematocrit, %	40 (28.3-48.5)	39.4 (26.6-49.2)	38.3 (25.8-49.1)	0.031
Platelet count, $10^3/mm^3$	274 (134-512)	271 (114-467)	248.5 (123-487)	0.112
MPV, fL	8.5 (7.2-10.6)	8.6 (7.1-10.5)	8.6 (7.2-10.2)	0.343
RDW,	13.9 (12.7-16.0)	14.1 (12.9-18.0)	14.3 (12.9-18.0)	0.001
PLR	348.3 (121.9-1036)	398.6 (134.5-1260)	398.4 (120.8-1325)	0.061
NLR	10.3 (2.6-39.7)	13.4 (3.6-52.6)	15.2 (3.2-52.0)	<0.001
dNLR	5.78 (1.55-15.5)	6.94 (2.0-20.3)	7.85 (1.82-19.2)	0.003
MLR	0.66 (0.2-2.0)	0.76 (0.2-2.5)	0.81 (0.21-3.0)	0.025
ELR	0 (0-0.3)	0 (0-0.36)	0 (0-0.5)	0.483
BLR	0 (0-0.11)	0 (0-0.1)	0 (0-0.16)	0.126
SII	2857.1 (556.5-10670.6)	3528 (884.4-13530)	3685 (693.5-14869.3)	0.005
SIRI	5.44 (0.96-19.87)	6.48 (1.34-34.2)	7.6 (1.22-36.7)	0.003
AISI	1254.2 (247.7-5950)	1732.9 (302.9-9500.1)	1956.8 (294.6-9911.2)	0.023
NHL	0.75 (0.18-3.09)	1.0 (2.8-3.94)	1.21 (0.26-4.12)	<0.001

AISI, aggregate index of systemic inflammation; *BLR*, basophil-to-lymphocyte ratio; *dNLR*, derived NLR; *ELR*, eosinophil-lymphocyte ratio; *MLR*, Monocyte to lymphocyte ratio; *MPV*, mean platelet volume; *NHL*, neutrophil-to-hemoglobin and lymphocyte; *NLR*, neutrophil to lymphocyte ratio; *PLR*, platelet to lymphocyte ratio; *RDW*, red blood cell distribution width; *SII*, systemic immune-inflammation index; *SIRI*, systemic inflammation response index. *WBC*, white blood cell count.
Statistical analyses were performed by Kruskal Wallis test comparing median (P_5-P_{95}).
p-value <0.05 was considered statistically significant.

Supplementary TABLE 3 | Hematological parameters and systemic inflammation indices according to IMV support and outcome in COVID-19

**Characteristics**	**IMV**			**Outcome**		
**Parameters**	**No (n = 196)**	**Yes (n = 352)**	***p*-value**	**Survival (n = 491)**	**Non-survival (n = 316)**	***p*-value**
WBC,10^3/mm^3	8.25 (3.9-16.5)	10.4 (5.3-19.6)	<0.001	9.5 (4.8-17.6)	10.8 (5.4-20.2)	<0.001
Neutrophil count, 10^3/mm^3	6.5 (2.7-13.8)	9.1 (4.3-17.3)	<0.001	8 (3.8-16.2)	9.7 (4.5-18.3)	<0.001
Lymphocyte count, 10^3/mm^3	0.7 (0.3-2.2)	0.6 (0.2-1.9)	0.002	0.7 (0.3-2.0)	0.6 (0.2-1.6)	<0.001
Monocyte count, 10^3/mm^3	0.5 (0.2-1.1)	0.5 (0.2-1.2)	0.042	0.5 (0.2-1.2)	0.5 (0.1-1.1)	0.156
Eosinophil, count 10^3/mm^3	0 (0-0.3)	0 (0-0.4)	0.004	0 (0-0.4)	0 (0-0.4)	0.835
Basophil count, 10^3/mm^3	0 (0-0.1)	0 (0-0.1)	0.420	0 (0-0.1)	0 (0-0.1)	0.602
Erythrocytes, 10^6/mm^3	4.8 (3.8-5.6)	4.3 (2.9-5.4)	<0.001	4.5 (3.0-5.5)	4.2 (2.7-5.4)	<0.001
Hemoglobin, gr/dL	14.5 (11.4-17)	13.1 (8.8-16.8)	<0.001	13.5 (9.3-16.8)	13.1 (8.3-16.9)	0.010
Hematocrit, %	42.3 (33.9-48.8)	38.6 (25.8-49.1)	<0.001	39.7 (27.9-48.9)	38.5 (25.2-49.3)	0.039
Platelet count, 10^3/mm^3	278 (145-485)	266 (115-485)	0.203	279 (145-500)	241.5 (95.5-456.5)	<0.001
MPV, fL	8.5 (7.1-10.3)	8.6 (7.2-10.4)	0.321	8.5 (7.1-10.2)	8.7 (7.2-10.6)	0.008
RDW	13.8 (12.7-16.7)	14.2 (13-18.2)	<0.001	14 (12.9-17.4)	14.4 (13-17.9)	0.001
PLR	386.6 (129.1-1036)	390 (126.4-1300)	0.094	379.5 (126.4-1196.6)	419 (130.0-1433.3)	0.112
NLR	8.3 (2.2-28.3)	14 (3.7-52.6)	<0.001	11 (2.9-42.5)	16.5 (4.5-62.0)	<0.001
dNLR	5.2 (1.5-14.0)	7.3 (2.1-20.2)	<0.001	6.0 (1.78-15.7)	8.3 (2.8-23.0)	<0.001
MLR	0.58 (0.20-1.75)	0.78 (0.22-2.7)	<0.001	0.66 (0.22-2.0)	0.83 (0.20-3.2)	0.001
ELR	0 (0-0.2)	0 (0-0.4)	0.004	0 (0-0.35)	0 (0-0.48)	0.476
BLR	0 (0-0.1)	0 (0-0.12)	0.422	0 (0-0.1)	0 (0-0.2)	0.425
SII	2317.7 (556.5-9873.5)	3608.9 (871-13816)	<0.001	3160.2 (688.5-12515.6)	4028.6 (897.8-15261)	<0.001
SIRI	3.89 (0.98-16.8)	7.0 (1.39-34.7)	<0.001	5.8 (1.1-26.3)	7.7 (1.5-41.9)	<0.001
AISI	1043.7 (230.6-4596.8)	1826.3 (306.9-9871.7)	<0.001	1527.2 (260.9-8312.5)	1839.2 (334.3-11295.1)	0.048
NHL	0.59 (0.14-2.16)	1.1 (0.29-4.1)	<0.001	0.83 (0.20-3.2)	1.3 (0.39-4.5)	<0.001

AISI, aggregate index of systemic inflammation; *BLR*, basophil-to-lymphocyte ratio; *dNLR*, derived NLR; *ELR*, eosinophil-lymphocyte ratio; *MLR*, Monocyte to lymphocyte ratio ; *MPV*, mean platelet volume; *NHL; NHL*, neutrophil-to-hemoglobin and lymphocyte; *NLR*, neutrophil to lymphocyte ratio; *PLR*, platelet to

lymphocyte ratio; *RDW*, red blood cell distribution width; *SII*, systemic immune-inflammation index; *SIRI*, systemic inflammation response index; *WBC*, white blood cell count.

Statistical analyses were performed by Mann Whitney U test comparing median (P_5-P_{95}).

p-value <0.05 was considered statistically significant.

FIGURE

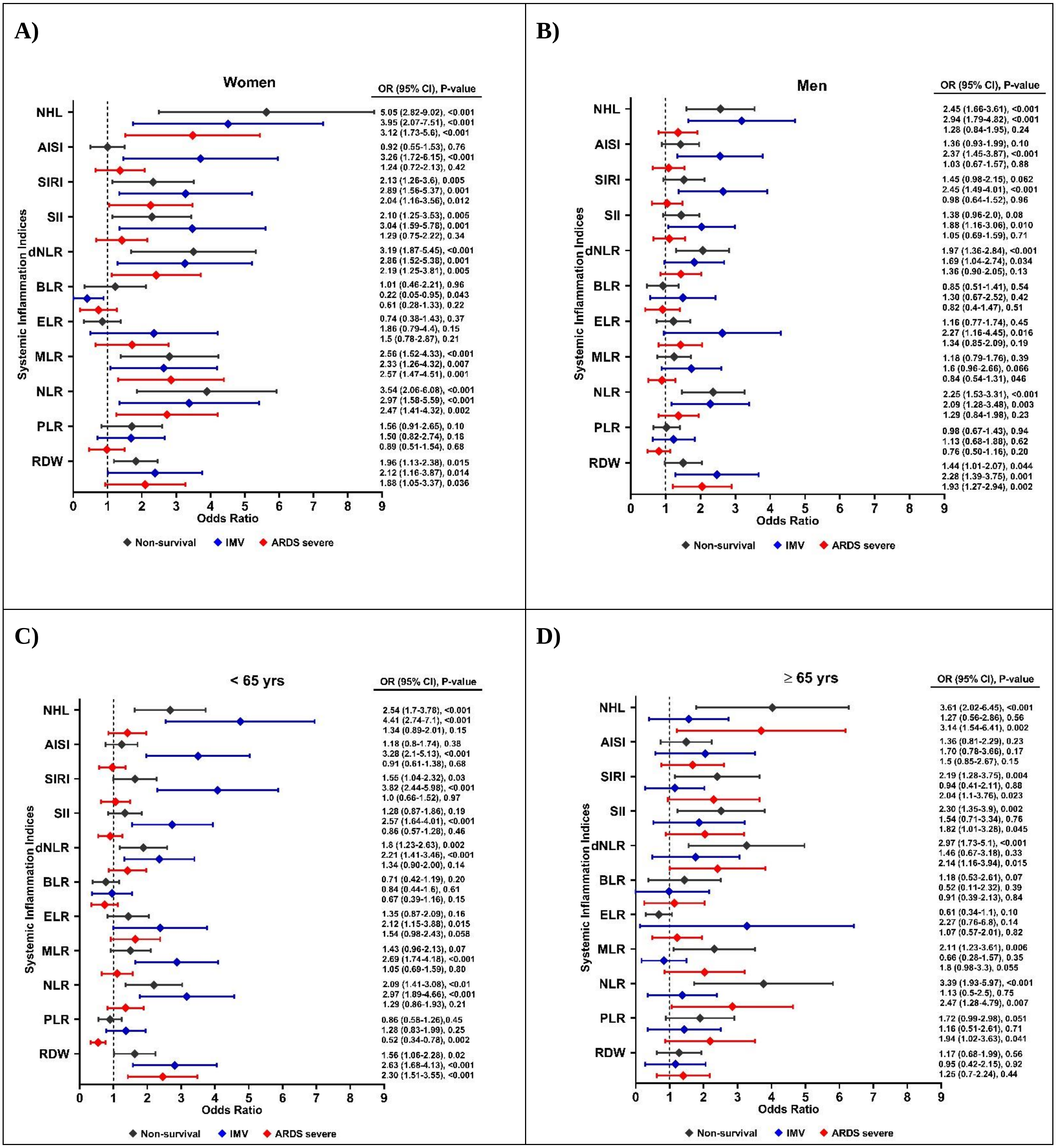
A)
Women
OR (95% CI), P-value
NHL
5.05 (2.82-9.02), <0.001
3.95 (2.07-7.51), <0.001
3.12 (1.73-5.6), <0.001
AISI
0.92 (0.55-1.53), 0.76
3.26 (1.72-6.15), <0.001
1.24 (0.72-2.13), 0.42
SIRI
2.13 (1.26-3.6), 0.005
2.89 (1.56-5.37), 0.001
2.04 (1.16-3.56), 0.012
SII
2.10 (1.25-3.53), 0.005
3.04 (1.59-5.78), 0.001
1.29 (0.75-2.22), 0.34
dNLR
3.19 (1.87-5.45), <0.001
2.86 (1.52-5.38), 0.001
2.19 (1.25-3.81), 0.005
BLR
1.01 (0.46-2.21), 0.96
0.22 (0.05-0.95), 0.043
0.61 (0.28-1.33), 0.22
ELR
0.74 (0.38-1.43), 0.37
1.86 (0.79-4.4), 0.15
1.5 (0.78-2.87), 0.21
MLR
2.56 (1.52-4.33), <0.001
2.33 (1.26-4.32), 0.007
2.57 (1.47-4.51), 0.001
NLR
3.54 (2.06-6.08), <0.001
2.97 (1.58-5.59), <0.001
2.47 (1.41-4.32), 0.002
PLR
1.56 (0.91-2.65), 0.10
1.50 (0.82-2.74), 0.18
0.89 (0.51-1.54), 0.68
RDW
1.96 (1.13-2.38), 0.015
2.12 (1.16-3.87), 0.014
1.88 (1.05-3.37), 0.036
Systemic Inflammation Indices
Odds Ratio
0 1 2 3 4 5 6 7 8 9
Non-survival IMV ARDS severe
B)
Men
OR (95% CI), P-value
NHL
2.45 (1.66-3.61), <0.001
2.94 (1.79-4.82), <0.001
1.28 (0.84-1.95), 0.24
AISI
1.36 (0.93-1.99), 0.10
2.37 (1.45-3.87), <0.001
1.03 (0.67-1.57), 0.88
SIRI
1.45 (0.98-2.15), 0.062
2.45 (1.49-4.01), <0.001
0.98 (0.64-1.52), 0.96
SII
1.38 (0.96-2.0), 0.08
1.88 (1.16-3.06), 0.010
1.05 (0.69-1.59), 0.71
dNLR
1.97 (1.36-2.84), <0.001
1.69 (1.04-2.74), 0.034
1.36 (0.90-2.05), 0.13
BLR
0.85 (0.51-1.41), 0.54
1.30 (0.67-2.52), 0.42
0.82 (0.4-1.47), 0.51
ELR
1.16 (0.77-1.74), 0.45
2.27 (1.16-4.45), 0.016
1.34 (0.85-2.09), 0.19
MLR
1.18 (0.79-1.76), 0.39
1.6 (0.96-2.66), 0.066
0.84 (0.54-1.31), 046
NLR
2.25 (1.53-3.31), <0.001
2.09 (1.28-3.48), 0.003
1.29 (0.84-1.98), 0.23
PLR
0.98 (0.67-1.43), 0.94
1.13 (0.68-1.88), 0.62
0.76 (0.50-1.16), 0.20
RDW
1.44 (1.01-2.07), 0.044
2.28 (1.39-3.75), 0.001
1.93 (1.27-2.94), 0.002
Systemic Inflammation Indices
Odds Ratio
0 1 2 3 4 5 6 7 8 9
Non-survival IMV ARDS severe
C)
< 65 yrs
OR (95% CI), P-value
NHL
2.54 (1.7-3.78), <0.001
4.41 (2.74-7.1), <0.001
1.34 (0.89-2.01), 0.15
AISI
1.18 (0.8-1.74), 0.38
3.28 (2.1-5.13), <0.001
0.91 (0.61-1.38), 0.68
SIRI
1.55 (1.04-2.32), 0.03
3.82 (2.44-5.98), <0.001
1.0 (0.66-1.52), 0.97
SII
1.28 (0.87-1.86), 0.19
2.57 (1.64-4.01), <0.001
0.86 (0.57-1.28), 0.46
dNLR
1.8 (1.23-2.63), 0.002
2.21 (1.41-3.46), <0.001
1.34 (0.90-2.00), 0.14
BLR
0.71 (0.42-1.19), 0.20
0.84 (0.44-1.6), 0.61
0.67 (0.39-1.16), 0.15
ELR
1.35 (0.87-2.09), 0.16
2.12 (1.15-3.88), 0.015
1.54 (0.98-2.43), 0.058
MLR
1.43 (0.96-2.13), 0.07
2.69 (1.74-4.18), <0.001
1.05 (0.69-1.59), 0.80
NLR
2.09 (1.41-3.08), <0.01
2.97 (1.89-4.66), <0.001
1.29 (0.86-1.93), 0.21
PLR
0.86 (0.58-1.26),0.45
1.28 (0.83-1.99), 0.25
0.52 (0.34-0.78), 0.002
RDW
1.56 (1.06-2.28), 0.02
2.63 (1.68-4.13), <0.001
2.30 (1.51-3.55), <0.001
Systemic Inflammation Indices
Odds Ratio
0 1 2 3 4 5 6 7 8 9
Non-survival IMV ARDS severe
D)
≥ 65 yrs
OR (95% CI), P-value
NHL
3.61 (2.02-6.45), <0.001
1.27 (0.56-2.86), 0.56
3.14 (1.54-6.41), 0.002
AISI
1.36 (0.81-2.29), 0.23
1.70 (0.78-3.66), 0.17
1.5 (0.85-2.67), 0.15
SIRI
2.19 (1.28-3.75), 0.004
0.94 (0.41-2.11), 0.88
2.04 (1.1-3.76), 0.023
SII
2.30 (1.35-3.9), 0.002
1.54 (0.71-3.34), 0.76
1.82 (1.01-3.28), 0.045
dNLR
2.97 (1.73-5.1), <0.001
1.46 (0.67-3.18), 0.33
2.14 (1.16-3.94), 0.015
BLR
1.18 (0.53-2.61), 0.07
0.52 (0.11-2.32), 0.39
0.91 (0.39-2.13), 0.84
ELR
0.61 (0.34-1.1), 0.10
2.27 (0.76-6.8), 0.14
1.07 (0.57-2.01), 0.82
MLR
2.11 (1.23-3.61), 0.006
0.66 (0.28-1.57), 0.35
1.8 (0.98-3.3), 0.055
NLR
3.39 (1.93-5.97), <0.001
1.13 (0.5-2.5), 0.75
2.47 (1.28-4.79), 0.007
PLR
1.72 (0.99-2.98), 0.051
1.16 (0.51-2.61), 0.71
1.94 (1.02-3.63), 0.041
RDW
1.17 (0.68-1.99), 0.56
0.95 (0.42-2.15), 0.92
1.25 (0.7-2.24), 0.44
Systemic Inflammation Indices
Odds Ratio
0 1 2 3 4 5 6 7 8 9
Non-survival IMV ARDS severe

E)

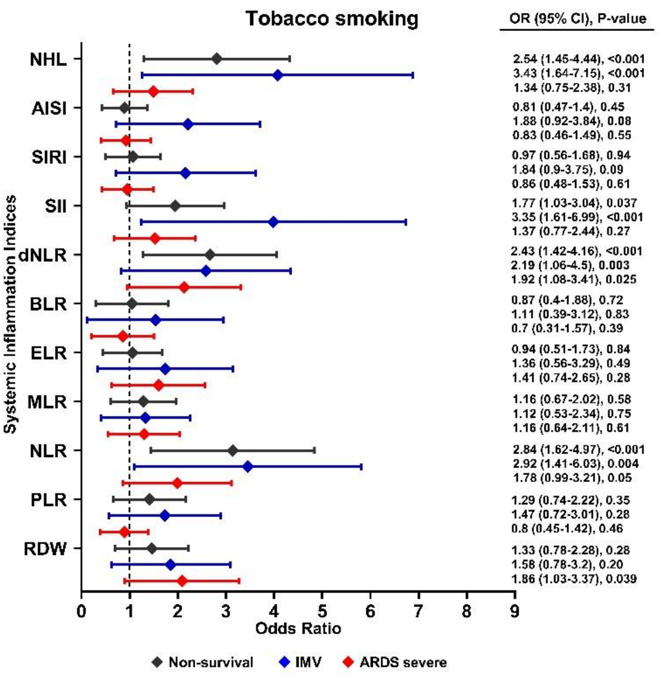

F)

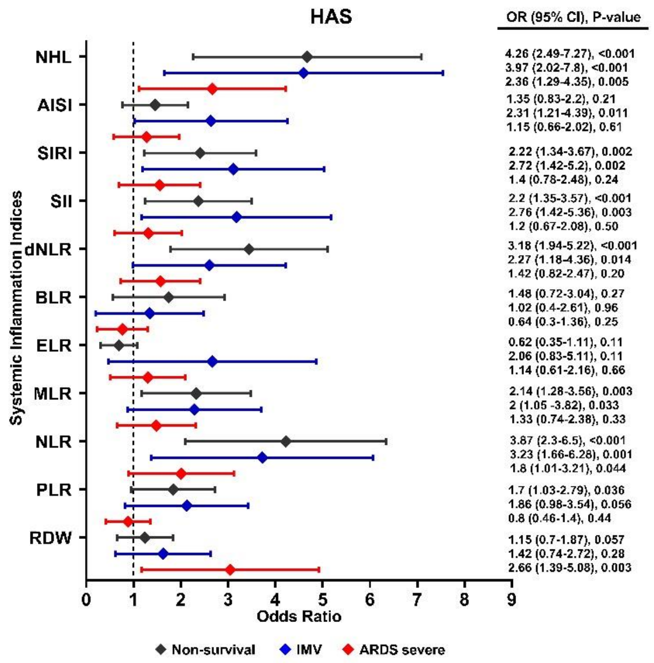

G)

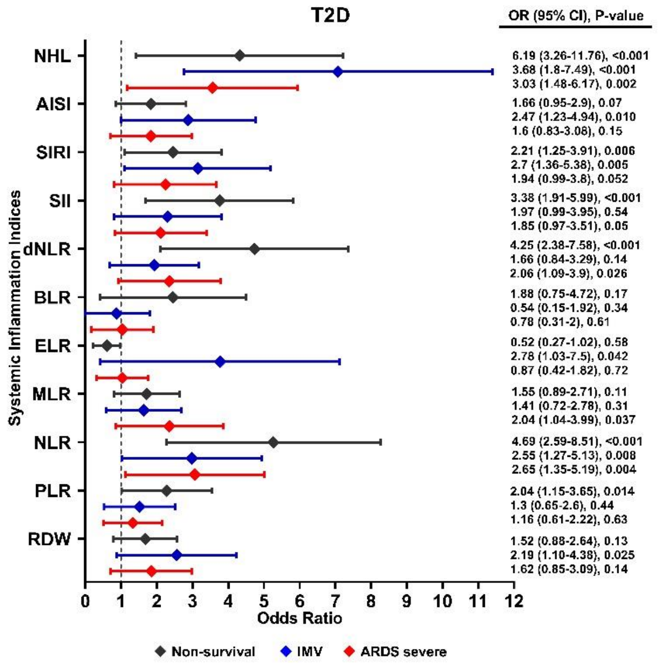

Supplementary Figure 1 | Association between systemic inflammation indices and severe COVID-19, IMV support, and non-survival by COVID-19 according to sex, age, and comorbidities presence in Mexican patients. (A) Women, (B) Men, (C) ≤65 yrs, (D) ≥65 yrs, (E) Tobacco smoking, (F) HAS, and (G) T2D. A logistic regression crude analysis. Significance was set at *p*-value <0.05

FIGURE

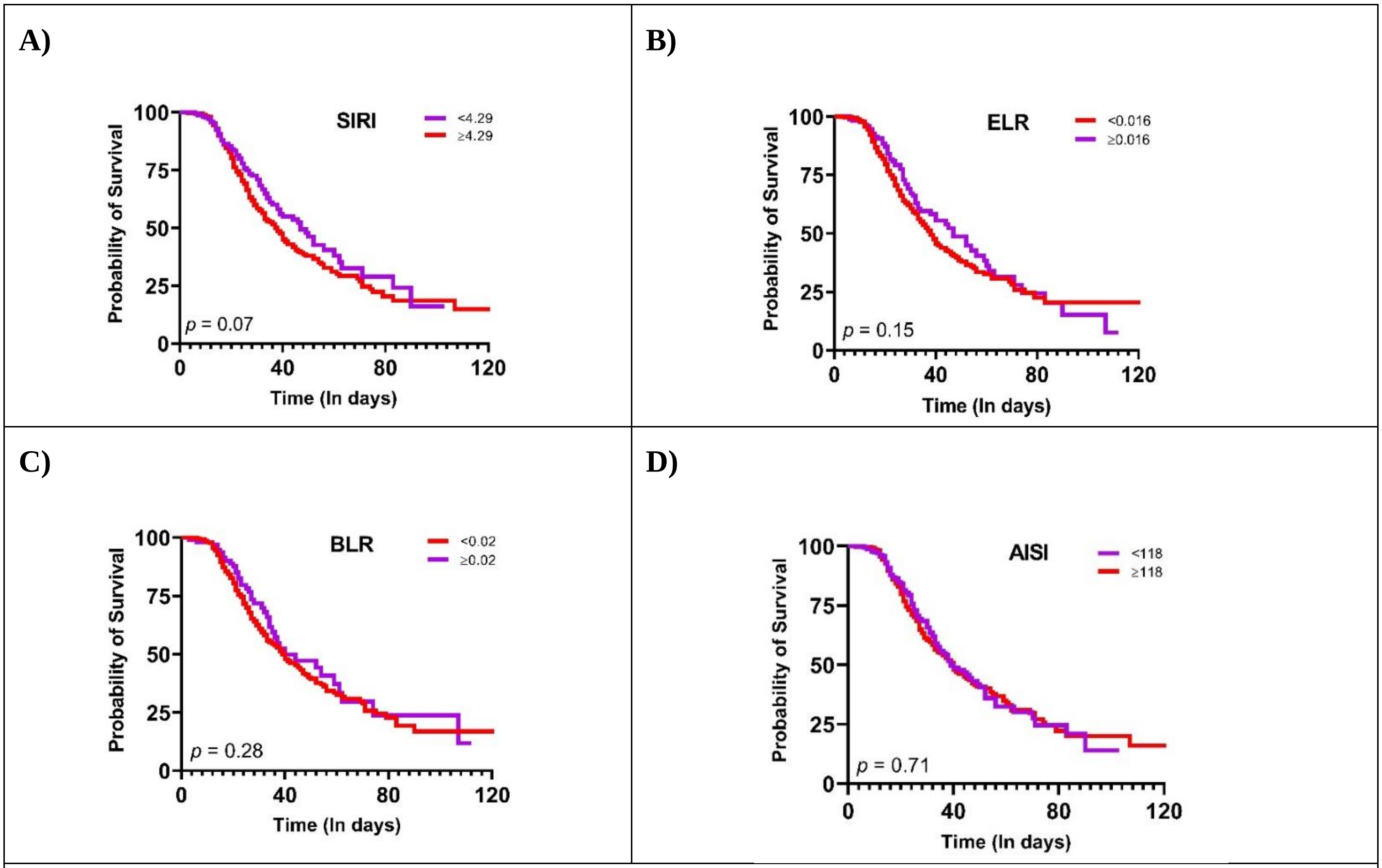

Supplementary Figure 2 | Kaplan–Meier survival curves during hospitalization of COVID-19 in Mexican patients with different cut-off values of the systemic inflammation indices not significantly related. (A) SIRI, (B) ELR, (C) BLR, (D) AISI. Statistical analyses were performed by the Kaplan-Meier method and compared using the Log-rank test. Significance was set at *p*-value <0.05.
